# Rothmund–Thomson Syndrome: novel pathogenic mutations and frequencies of variants in the *RECQL4* and *USB1 (C16orf57)* gene

**DOI:** 10.1002/mgg3.209

**Published:** 2016-02-24

**Authors:** Aude‐Annick Suter, Peter Itin, Karl Heinimann, Munaza Ahmed, Tazeen Ashraf, Helen Fryssira, Usha Kini, Pablo Lapunzina, Peter Miny, Mette Sommerlund, Mohnish Suri, Signe Vaeth, Pradeep Vasudevan, Sabina Gallati

**Affiliations:** ^1^ Division of Human GeneticsDepartment of PaediatricsInselspitalUniversity of BernCH‐3010BernSwitzerland; ^2^ Department of Clinical ResearchUniversity of BernCH‐3010BernSwitzerland; ^3^ Department of DermatologyUniversity of BaselBaselSwitzerland; ^4^ Medical GeneticsUniversity Hospital BaselBaselSwitzerland; ^5^ Wessex Clinical Genetics ServiceUniversity Hospital SouthamptonSouthamptonUK; ^6^ Department of Clinical GeneticsGuys HospitalLondonUK; ^7^ Department of Clinical GeneticsAgia Sophia Children's HospitalAthensGreece; ^8^ Department of Clinical GeneticsOxford University Hospitals NHS TrustOxfordUK; ^9^ INGEMM, Institute of Medical and Molecular GeneticsUniversity Hospital La Paz, IdiPAZ‐CIBERERMadridSpain; ^10^ Department of DermatologyAarhus University HospitalAarhusDenmark; ^11^ Departement of Clinical GeneticsNottingham University Hospitals NHS TrustCity Hospital CampusNottinghamUK; ^12^ Department of Clinical GeneticsAarhus University HospitalAarhusDenmark; ^13^ Department of Clinical GeneticsUniversity Hospitals of Leicester NHS TrustLeicester Royal InfirmaryLeicesterUK

**Keywords:** Poikiloderma with neutropenia, *RECQL4* gene, Rothmund–Thomson Syndrome, *USB1* (*C16orf57)* gene

## Abstract

**Background:**

Poikiloderma is defined as a chronic skin condition presenting with a combination of punctate atrophy, areas of depigmentation, hyperpigmentation and telangiectasia. In a variety of hereditary syndromes such as Rothmund–Thomson syndrome (RTS), Clericuzio‐type poikiloderma with neutropenia (PN) and Dyskeratosis Congenita (DC), poikiloderma occurs as one of the main symptoms. Here, we report on genotype and phenotype data of a cohort of 44 index patients with RTS or related genodermatoses.

**Methods:**

DNA samples from 43 patients were screened for variants in the 21 exons of the *RECQL4* gene using PCR, SSCP‐PAGE analysis and/or Sanger sequencing. Patients with only one or no detectable mutation in the *RECQL4* gene were additionally tested for variants in the 8 exons of the *USB1 (C16orf57)* gene by Sanger sequencing. The effect of novel variants was evaluated by phylogenic studies, single‐nucleotide polymorphism (SNP) databases and in silico analyses.

**Results:**

We identified 23 different *RECQL4* mutations including 10 novel and one homozygous novel *USB1 (C16orf57)* mutation in a patient with PN. Moreover, we describe 31 *RECQL4* and 8 *USB1* sequence variants, four of them being novel intronic *RECQL4* sequence changes that may have some deleterious effects on splicing mechanisms and need further evaluation by transcript analyses.

**Conclusion:**

The current study contributes to the improvement of genetic diagnostic strategies and interpretation in RTS and PN that is relevant in order to assess the patients' cancer risk, to avoid continuous and inconclusive clinical evaluations and to clarify the recurrence risk in the families. Additionally, it shows that the phenotype of more than 50% of the patients with suspected Rothmund–Thomson disease may be due to mutations in other genes raising the need for further extended genetic analyses.

## Introduction

Rothmund–Thomson syndrome (RTS, OMIM #268400) is a rare, heterogeneous autosomal recessive genodermatosis due to biallelic mutations in the *RECQL4* gene (OMIM #603780) in about 66% of patients (Cabral et al. 2008; Larizza et al. 2010). The exact prevalence of RTS is still unknown, and only around 400 cases have been described in the literature so far (Larizza et al. 2013, 2010). The diagnostic hallmark of RTS is chronic poikiloderma (Larizza et al. 2010) that arises during the first 2 years of life, even though late onset of poikiloderma has also been reported (Kumar et al. 2007). Other clinical features include skeletal abnormalities (e.g., short stature, frontal bossing, saddle nose, short fingers, radial ray defects), alopecia, sparse or missing eyelashes and/or eyebrows, photosensitivity, juvenile cataracts, premature aging and a predisposition to malignancies, especially to osteosarcoma and skin cancers (squamous and basal cell carcinomas) (Larizza et al. 2010; Wang et al. 2001; Zils et al. 2015). On the basis of clinical and molecular analysis, two forms of RTS have been characterized: RTS type I, presenting with typical poikiloderma and juvenile cataracts without *RECQL4* mutations (Siitonen et al. 2009; Wang et al. 2001) and RTS type II with poikiloderma and an increased risk for osteosarcoma caused by deleterious mutations in *RECQL4* (Larizza et al. 2013; Wang et al. 2003). Differential diagnoses of RTS are dyskeratosis congenita (DC) and poikiloderma with neutropenia (PN) (all of them causing poikiloderma at young age) as well as other rare genodermatoses with a telangiectatic component (Bloom syndrome, Werner syndrome) and other diseases with mutations in the *RECQL4* gene such as RAPADILINO and Baller–Gerold syndrome (BGS) (Larizza et al. 2010).

The *RECQL4* gene is located on chromosome 8q24.3 spanning 21 exons and belongs to the protein family of DNA helicases that are important in maintaining genomic integrity (Croteau et al. 2012b; Lu et al. 2014). It encodes the ATP‐dependent DNA helicase Q4 (RecQ protein‐like 4), a 133‐kDa protein consisting of 1208 amino acids. RECQL4 participates in base excision repair, nucleotide excision repair, homologous recombination and in the initiation of DNA replication (Croteau et al. 2012b; Liu 2010). Moreover, it plays a role in telomere (Ghosh et al. 2012) as well as in mitochondrial DNA maintenance (Croteau et al. 2012a) and interacts with the tumor suppressor p53 (De et al. 2012).

Mutations in the *USB1 (C16orf57)* (U6 snRNA biogenesis 1, OMIM #613276) gene give rise to autosomal‐recessive Clericuzio‐type poikiloderma with neutropenia (PN, OMIM #604173), characterized by early‐onset poikiloderma (appearing in the first year of life), nail abnormalities, palmoplantar hyperkeratosis, skeletal defects as well as noncycling neutropenia and recurrent sinopulmonary infections (Colombo et al. 2012). The rash is eczematous and starts peripherally before spreading centrally to the trunk and face. Furthermore, individuals with this very rare disorder may present with craniofacial dysmorphism, carious teeth and postnatal growth delay (Farruggia et al. 2014).

The *USB1* gene is localized on 16q21, contains 7 exons spanning 22 kb and encodes a 265‐amino acid protein with 5 helical domains that is highly conserved among vertebrates. Although the function of the USB1 protein remains still unclear, there is evidence that it is essential for the processing and stability of U6 small nucleolar RNA with a pivotal role in RNA splicing (Mroczek et al. 2012; Shchepachev et al. 2012).

Here, we present genotype and phenotype data of a cohort of 44 index patients with clinically suspected RTS or related genodermatoses. We delineate the spectrum of pathogenic mutations including 10 novel *RECQL4* mutations and one homozygous novel *USB1* mutation as well as of 31 and 8 different gene variants respectively.

## Patients and Methods

### Ethical compliance

The local Swiss Ethics Committee on research involving humans approved this study.

### Patient cohort

A total of 44 (22 females, 22 males; age range 0.2–39 years) unrelated, ethnically diverse patients with clinical suspicion of RTS or related genodermatoses and referred to our laboratory from 2005 to 2014 were included in this study. Appropriate informed consent for genetic testing was obtained from the patients or their parents.

### Methods

DNA samples from 43 patients (numbered consecutively from 1 through 43) were screened for variants in the 21 exons (including boundaries for exon/intron 3, 4, 6, 9, 12 and entire sequences of intron 1, 2, 5, 7, 8, 11, 13–20) of the *RECQL4* gene using PCR, SSCP‐PAGE analysis and/or Sanger sequencing as described before (Gallati et al. 2009). Additionally, patients with only one or no detectable mutation in the *RECQL4* gene as well as patient No 44, who presented with a classic PN phenotype, were tested for variants in the coding region of the *USB1* gene (7 exons including exon/intron boundaries) by Sanger sequencing. Primer sequences and PCR conditions are available upon request. Mutation positions are given according to the Reference sequences for *RECQL4* (GenBank NM_004260.3) and *USB1* (GenBank NM_024598.3), numbering starting at the A of the ATG initiation codon, and nomenclature is according to the Human Genome Variation Society (www.hgvs.org/mutnomen/). The effect of novel variants was evaluated by phylogenic studies, single nucleotide polymorphism (SNP) databases (1000 genomes (http://browser.1000genomes.org/index.html), Exome Variant Server (http://evs.gs.washington.edu/EVS/) and ExAC Browser (http://exac.broadinstitute.org)) and in silico analyses using MutPred (http://mutpred.mutdb.org), PredictSNP (http://loschmidt.chemi.muni.cz/predictsnp), SNPs&GO (http://snps-and-go.biocomp.unibo.it/snps-and-go/), PolyPhen‐2 (http://genetics.bwh.harvard.edu/pph2/) and Provean (http://provean.jcvi.org). The variant data and phenotypes were registered and are available at http://databases.lovd.nl/shared/genes/RECQL4 and http://www.ncbi.nlm.nih.gov/clinvar/?term=USB1%5Bgene%5D. Statistical analyses have been performed using chi‐squared statistic and Fisher's exact test.

## Results

### *RECQL4* analyses

**Figure 1:**
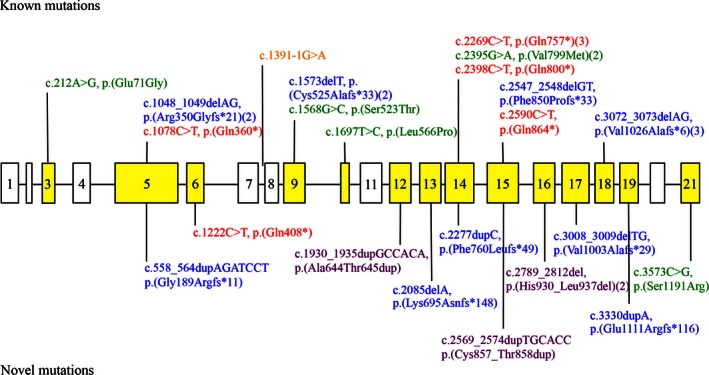
Scheme of the *RECQL4* Gene. Boxes and introns indicate exons by lines. Yellow identifies the exons with identified mutations, white the exons without mutations. Red characters = nonsense mutations, blue = frameshift mutations, purple = inframe mutations, green = missense mutations. *RECQL4* GenBank NM_004260.3.

Figure 1 summarizes the *RECQL4* mutation spectrum of our patient cohort. Overall, 23 different *RECQL4* mutations were identified in 18 (42%) out of 43 index patients (patient No 44 was solely tested for mutations in the *USB1* gene based on high clinical suspicion of PN). Two patients were found to be homozygous due to parental consanguinity, 10 patients showed heterozygosity for two and one patient for three different mutations and in 5 patients only one deleterious sequence change was detectable. As illustrated in Figure 1, 13 of the identified mutations are already listed in the Human Gene Mutation Database (HGMD, http://www.biobase-international.com) and/or reported in publications, 5 of them presenting as recurrent sequence changes. The remaining 10 sequence variants including 5 frameshift, 3 in‐frame, 1 nonsense and 1 missense mutations have not been reported so far. In summary, the *RECQL4* mutation spectrum of our patient cohort spreads almost all over the gene with the exception of exons 1, 2, 4, 7, 8, 11, and 20 and is represented by 1 splicing, 7 nonsense, 13 frameshift, 4 in‐frame, and 6 missense mutations.

In addition to primarily disease causing mutations, we detected 31 different variants in 8 exons and 12 introns of the *RECQL4* gene including 14 well known polymorphisms, 10 likely benign variants and 7 variants of unknown significance (VUS) (Table 1). Four intronic variants (c.213+10C>G, c.1391‐6C>A, c.1878+22G>A, c.2464‐21C>A) have not been described before and are not listed in any of the databases that have been checked for these positions. Allele frequencies of our patient cohort were compared with data from the SNP databases 1000 genomes, Exome Variant Server and ExAC Browser and significant differences between frequences were found for 3 exonic and 4 intronic variants (Table 1).

**Table 1:** Allele frequencies of *RECQL4* and *USB1* (*C16orf57*) variants

*RECQL4* exon (E) intron (I)	dpSNP ID	Variant	Frequencies %, *n* = 86		Ensembl frequencies %, 1000 genomes		ExAC frequencies %, *n* > 100,000		Exome Variant Server frequencies %, *n* > 12,000		*P* value
5′UTR	rs35667555	c.‐37C>A	C 98.8	A 1.2	C 99.6	A 0.4	C 98.7	A 1.3	–	–	n.s.
E03	rs2306386	*c.132A>G (p.=)*	A 74.4	G 25.6	A 42.5	G 57.5	A 44.5	G 55.5	A 44.9	G 55.1	<0.0001
	rs35198096	c.161A>G (p.(Gln54Arg))	A 98.8	G 1.2	A 99.1	G 0.9	G 99.5	G 0.5	A 99.5	G 0.5	n.s.
I03	rs565610567 (C/A/T)	**c.213+10C>G** a	C 98.8	G 1.2	–	–	–	–	–	–	–
	rs2721189	*c.213+82G>T*	G 80.2	T 19.8	G 5.2	T 94.8	–	–	–	–	<0.0001
E04	rs200516441	c.275C>T (p.(Ser92Phe))	C 97.7	T 2.3	C 99.0	T 1.0	C 99.8	T 0.2	C 99.5	T 0.5	n.s.
	rs4251688	c.309G>A (p.=)	G 98.8	A 1.2	G 99.6	A 0.4	G 99.3	A 0.7	G 99.6	A 0.4	n.s.
I04	rs35058172	c.355‐24G>C	G 98.8	C 1.2	G 98.8	C 1.2	G 99.6	C 0.4	G 99.1	C 0.9	n.s.
E05	rs34159914	c.543G>A (p.=)	G 97.7	A 2.3	G 99.9	A 0.1	G 99.6	A 0.4	G 99.6	A 0.4	n.s.
	rs4244613	*c.738C>T (p.=)*	C 79.1	T 20.9	C 63.0	T 37.0	C 56.1	T 43.9	C 66.1	T 33.9	0.011
	rs4244612	*c.801G>C (p.(Glu267Asp))*	G 80.2	C 19.8	G 55.2	C 44.8	G 56.2	C 43.8	G 60.8	C 39.2	0.0002
I06	rs34437789	c.1258+6A>T	A 96.5	T 3.5	A 98.5	T 1.5	A 97.5	T 2.5	A 97.4	T 2.6	n.s.
	rs4251689	*c.1258+18G>A*	G 84.9	A 15.1	G 59.9	A 40.1	G 52.8	A 47.2	G 63.5	A 36.5	<0.0001
I07	rs766263452 (C/T)	**c.1391‐6C>A** a	C 98.8	A 1.2	–	–	–	–	–	–	–
E09	rs754735053	c.1568G>C (p.(Ser523Thr))^a^	G 98.8	C 1.2	–	–	G 99.97	C 0.03	–	–	–
I09	rs4244611	c.1621‐15C>T	C 72.1	T 27.9	C 60.5	T 39.5	C 52.0	T 48.0	C 64.9	T 35.1	n.s.
I10	rs35876881	c.1704+9C>T	C 97.7	T 2.3	C 99.7	T 0.3	C 99.8	T 0.2	C 99.7	T 0.3	n.s.
I11		**c.1878+22G>A** a	G 98.8	A 1.2	–	–	–	–	–	–	–
	rs35126141	c.1879‐15C>A	C 97.7	A 2.3	C 98.9	A 1.1	C 99.7	A 0.3	C 99.1	A 0.9	n.s.
I13	rs200942592	c.2201‐8C>T^a^	C 98.8	T 1.2	–	–	C 99.99	T 0.01	–	–	n.s.
E14	rs35215952	c.2238G>A (p.=)	G 98.8	A 1.2	G 99.0	A 1.0	G 99.6	A 0.4	G 99.2	A 0.8	n.s.
	rs34293591	c.2395G>A (p.(Val799Met))	G 98.8	A 1.2	G 99.4	A 0.6	G 98.2	A 1.8	G 98.3	A 1.7	n.s.
I14		**c.2464‐21C>A** a	C 98.8	A 1.2	–	–	–	–	–	–	–
E17	rs4251691	c.3014G>A (p.(Arg1005Gln))	G 66.3	A 33.7	G 61.6	A 38.4	G 53.9	A 46.1	G 65.8	A 34.2	n.s.
I18	rs4244610	*c.3236+13C>T*	C 70.9	T 29.1	C 56.7	T 43.3	C 49.4	T 50.6	C 60.7	T 39.3	0.0211
	rs780188311	c.3237‐20T>G^a^	T 98.8	G 1.2	–	–	>T 99.99	<G 0.01	–	–	–
I19	rs756627	c.3393+8C>T	C 70.9	T 29.1	C 59.9	T 40.1	C 53.3	T 46.7	C 65.0	T 35.0	n.s.
	rs4251692	c.3393+9A>G	A 97.7	G 2.3	A 99.3	G 0.7	A 97.7	G 2.3	A 98.7	G 1.3	n.s.
E20	rs61755066	c.3435G>C (p.(Gln1145His))	G 98.8	C 1.2	G 99.9	C 0.1	G 99.7	C 0.3	G 99.7	C 0.3	n.s.
I20	rs2279243	*c.3502*+*24G>A*	G 81.6	A 18.6	G 60.7	A 39.3	G 55.6	A 46.4	G 65.4	A 34.6	0.0012
E21	rs201384843	c.3609C>T (p.=)	C 97.7	T 2.3	C 99.9	T 0.1	C 99.7	T 0.3	C 99.8	T 0.2	n.s.

*USB1* exon (E) intron (I)	dpSNP ID	Variant	Frequencies %, *n* = 62		Ensembl frequencies %, 1000 genomes		ExAC frequencies %, *n* > 100,000		Exome Variant Server frequencies %, *n* > 12,000		*P* value
E01	rs3743559	c.42C>A (p.=)	C 93.6	A 6.4	C 93.9	A 6.1	C 89.8	A 10.2	–	–	n.s.
I01	rs3743561	c.99‐32A>T	A 96.8	T 3.2	A 93.9	T 6.1	A 93.0	T 7.0	–	–	n.s.
	rs18714585	c.99‐34T>A	T 96.8	A 3.2	T 93.9	A 6.1	T 93.9	A 6.1	–	–	n.s.
	rs3743560	c.99‐36G>A	G 30.7	A 69.3	G 43.2	A 56.8	G 43.2	A 56.8	–	–	n.s.
I04	rs4784022	*c.504‐60C>T*	C 83.9	T 16.1	C 50.2	T 49.8	–	–	–	–	<0.0001
I06	rs16959640	c.693+15C>T	C 96.8	T 3.2	C 91.1	T 8.9	C 97.2	T 2.8	–	–	n.s.
I06	rs12102539	c.694‐114G>A	G 96.8	A 3.2	G 98.3	A 1.7	–	–	–	–	n.s.
E07	rs16959641	c.748C>G (p.(Gln250Glu))	C 96.8	G 3.2	C 94.0	G 6.0	C 93.0	G 7.0	–	–	n.s.

n.s., not significant.

GenBank accession numbers: *RECQL4* NM_004260.3, *USB1* NM_024598.3.

Bold characters: novel variants identified in this study.

Italic characters: significant (*P* values < 0.05, based on two‐sided comparisons) differences in allele frequencies.

a Variant of unknown significance (VUS).

Clinical data from 12 out of 13 patients with two mutations identified in the *RECQL4* gene are summarized in Table 2. From one patient no clinical data were available. Besides poikiloderma or other skin lesions (100%), growth retardation and skeletal manifestations are the second (83%) and third (75%) most common features consistent with the diagnosis of RTS type II. The only patient with two missense mutations shows a much milder phenotype than all the other patients presenting with poikiloderma as the sole symptom.

**Table 2:** Clinical data from 12 index patients presenting with two *RECQL4* mutations

Patient no.	Sex	Age (y)	Mutation	Nature of mutation	Exon Intron	Skin	Hair, nails, teeth	Eyes	Skeleton	Growth	Develop‐ment	Gastro‐intestinal	Cancer
5	F	1.5	c.161A>G	Missense	E03	1, 2, 3	10, 11, 12	–	16, 21	23, 24	26, 27	–	–
			**c.2569_2574dup**	Inframe	E15								
7	F	4.8	c.1697T>C	Missense	E10	7	–	–	18, 19	25	–	–	–
			**c.3330dupA**	Frameshift	E19								
8	F	13.0	c.2269C>T	Nonsense	E14	1, 2, 3, 5	8, 9, 10, 12	13	16, 18, 19	23, 25	27	–	–
			c.2547_2548delGT	Frameshift	E15								
9	M	7.2	c.212A>G	Missense	E03	1	–	–	–	–	–	–	–
			**c.3573C>G**	Missense	E21								
12	F	2.0	c.1391‐1G>A	Splicesite	I07	1	9,12	–	18	23, 24, 25	28	29	33
			**c.2085delA**	Nonsense	E13								
13	F	5.0	c.1048_1049delAG	Frameshift	E05	1	–	–	15, 17, 18, 19	–	–	–	–
			c.2398C>T	Nonsense	E14								
14	M	8.0	c.1573delT	Frameshift	E09	1, 2, 3, 4, 6	8, 9	–	–	25	26, 27	30	–
			**c.1930_1935dup**	Inframe	E12								
15	F	39.0	c.1078C>T	Nonsense	E05	1, 2, 4, 5, 6	8, 9	14	16, 18, 20	23, 25	–	–	31, 32
			**c.1222C>T**	Nonsense	E06								
16	M	1.7	**c.2789_2812del**	Inframe	E16	7	–	–	22	25	–	30	–
			**c.2789_2812del**	Inframe	E16								
17	M	10.8	c.3072_3073delAG	Frameshift	E18	1, 6	9	–	20	25	–	–	–
			c.3072_3073delAG	Frameshift	E18								
18	M	15.0	**c.558_564dup**	Frameshift	E05	1	–	–	–	25	–	–	–
			c.3072_3073delAG	Frameshift	E18								
19	M	1.8	**c.1568G>C**;c.1573delT	Frameshift	E09	2	8, 9	–	18, 19	24	–	30	–
			c.2269C>T	Nonsense	E14								

F, female; M, male; y, age at molecular genetic diagnosis in years; E, exon; I, intron.

Skin: 1, poikiloderma; 2, typical cutaneous rash (arms, face, legs); 3, erythema; 4, photosensitivity; 5, punctate atrophy; 6, hyperkeratotic zones; 7, multiple café‐au‐lait spots.

Hair, nails, teeth: 8, alopecia; 9, sparse eyebrows and/or eyelashes; 10, dystrophic nails; 11, delayed dentition; 12, hypoplastic teeth, microdontia.

Eyes: 13, eyelid coloboma; 14, photophobia.

Skeleton: 15, premature suture closure; 16, saddle nose; 17, congenital anomalies of the spine; 18, abnormalities of the long bones; 19, abnormally shaped thumbs; 20, osteopenia; 21, joint pain; 22, skeletal lesions.

Growth: 23, low birth weight; 24, failure to thrive; 25, linear growth deficiency, microsomia.

Development: 26, intellectual disability; 27, delayed speech development, 28 delayed motor development.

Gastrointestinal findings: 29, nutritional problems, gastrointestinal symptoms during infancy; 30, chronic/intermittent diarrhoea.

Cancer: 31, squamous cell carcinoma; 32, basal cell carcinoma, 33 osteosarcoma.

Bold characters: novel variants identified in this study.

### *USB1* analyses

Sequencing of the *USB1* gene in 31 patients with only one or no detectable *RECQL4* mutation identified eight known polymorphisms (Table 1), but no pathogenic mutation. However, in the only patient presenting with typical clinical symptoms of PN we detected homozygosity for a novel frameshift mutation c.334_335dupC (p.(Arg112Profs*31)) in exon 3 leading to a premature stop codon before the second H‐X‐S motif (Colombo et al. 2012). The phenotype of this patient was characterized by poikiloderma, neutropenia, short stature, hypogonadotropic hypogonadism (on hormonal replacement therapy) and noncaseating granuloma involving the lungs with interstitial lung disease and bronchiectasis.

## Discussion

To date, 76 different mutations of the *RECQL4* gene have been reported in the Human Gene Mutation Database (HGMD). Our study adds another 10 mutations that have not been described so far (Fig. 1). The nonsense mutation c.1222C>T (p.(Gln408*)) as well as the four frameshift mutations c.558_564dupAGATCCT (p.(Gly189Argfs*11)), c.2085delA (p.(Lys695Asnfs*148)), c.2277dupC (p.(Phe760Leufs*49)) and c.3008_3009delTG (p.(Val1003Alafs*29)) are assumed to result in a truncated protein, whereas the frameshift mutation c.3330dupA (p.(Glu1111Argfs*116)) causes an elongation of the gene product by 17 amino acids. The three in‐frame mutations c.1930_1935dupGCCACA (p.(Ala644Thr645dup)), c.2569_2574dupTGCACC (p.(Cys857_thr858dup)) and c.2789_2812del24 (p.(His930_Leu937del)) have been predicted to be deleterious by the software tool Provean. The results from different in silico analyses were somewhat conflicting for the missense mutation p.(Ser1191Arg). PredictSNP and MutPred predict this change to be deleterious, whereas PolyPhen‐2 and SNPs&GO propose a neutral state. Five of these novel mutations (4 truncating, 1 in‐frame) are localized before or in the conserved helicase domain (exons 8–14) shared by the other RecQ helicases and are thought to destroy or disturb the DNA helicase activity of RECQL4. The remaining five mutations (2 frameshift, 2 nonsense, 1 missense) downstream of the helicase domain may result in a loss or reduction of the RECQL4 repair function and maintenance of genome integrity. However, further studies such as transcript analyses and functional experiments are needed to characterize the effect of these novel *RECQL4* mutations.

In our study, we identified 18 patients (42%) with *RECQL4* mutations confirming the diagnosis of RTS II. One patient presented with three mutations, the frameshift mutation c.1573delT (p.(Cys525Alafs*33)) in *cis* with the amino acid substitution c.1568G>C (p.(Ser523Thr)) and the nonsense mutation c.2269C>T (p.(Gln757*)) in *trans*. The c.1568G>C (p.(Arg112Profs*31)Ser523Thr) variant is not yet listed in the SNP database but has been described in association with c.1573delT (p.(Cys525Alafs*33)) by other authors (Siitonen et al. 2009). In five patients only one *RECQL4* mutation was detectable suggesting that the second mutation has been missed by gene sequencing such as large deletions/duplications or is localized in gene regions that have not been analyzed (e.g., promoter, introns 3, 4, 6, 9, 12). As Piard et al. (2015) identified no large rearrangements in 29 patients with suspected RTS and only one *RECQL4* mutation or negative *RECQL4* sequencing by array‐CGH analysis, we believe that large deletions or duplications may occur rarely in RTS patients and that the focus of further analyses should be on promoter and intronic mutations as well as on other candidate genes.

Because PN is a possible differential diagnosis in the presence of poikiloderma, we sequenced the *USB1* gene in all patients with only one or no *RECQL4* mutation and in one patient with poikiloderma and neutropenia. We detected homozygosity for the novel mutation c.334_335dupC (p.(in the latter, but no pathogenic mutation in the other patients leading to the conclusion that mainly patients with a phenotype of poikiloderma and neutropenia are candidates for *USB1* mutation screening. In other respects, there is no distinct genotype‐phenotype correlation, particularly not with regard to specific mutations. In summary, we can state from Table 2 that the patient the with two missense mutations (9) tends to a milder phenotype with the only symptom of poikiloderma at the time of genetic analysis and that patients with two truncating mutations (No 8, 12, 13, 15, 17, 18, 19) show all skin, skeletal and/or growth abnormalities. In addition, patient No 15, carrying the novel nonsense mutation c.1222C>T (p.(Gln408*)) and the known nonsense mutation c.1078C>T (p.Gln360*)), has developed several squamous cell carcinomas (SCC) and basal cell carcinoma.

Besides of pathogenic mutations, we identified 31 *RECQL4* and 8 *USB1* variants and found statistically significant differences for seven (c.132A>G, c.213+82G>T, c.738C>T (p.=), c.801G>C (p.=), c.1258+18G>A, c.3236+13C>T, c.3502+24G>A) and one (c.504‐60C>T) of them respectively compared to population frequencies of Exome Variant Server and 1000 genomes databases (Table 1). The relevance of these findings is not yet clear. A disease association of these variants has been excluded as our patient cohort carries more wildtype alleles than the populations of the databases. This phenomenon needs further evaluation in order to determine if it is just the result of a very small patient number or if it is a fact either representing different ethnic background or even a protective effect of specific *RECQL4* and/or *USB1* sequence changes. Moreover, we detected four novel intronic *RECQL4* variants, one of them (c.1878+22G>A) in a patient with one truncating mutation and no second known mutation. This variant is worth testing on transcript level, as the *RECQL4* gene is known to have a specific splicing pattern based on the short size of 13 of its introns (Colombo et al. 2014).

The current study presents novel mutations and summarizes neutral variants of the *RECQL4* and *USB1* genes contributing to the improvement of data interpretation and diagnostic strategies in patients with suspicion of the very rare genodermatoses Rothmund–Thomson disease and poikiloderma with neutropenia. In case of unclear or negative findings gene sequencing should be accompanied by transcript analysis that, however, requires EBV‐transformed lymphoblastoid cell lines, cell cultures or fresh tissue for RNA isolation. The identification and characterization of *RECQL4* mutations is relevant in order to assess the patients' cancer risk, to avoid extensive and inconclusive clinical evaluations and to clarify the recurrence risk in the families. Our findings show, however, that the phenotype of more than 50% of the patients with suspected Rothmund–Thomson disease may be due to mutations in other genes raising the need for further extended genetic analyses.

## Conflict of Interest

The authors declare no conflict of interest.
